# Ethnic and Regional Differences in Prevalence and Correlates of Chronic Diseases and Risk Factors in Northern Canada

**Published:** 2009-12-15

**Authors:** Lisa M. Lix, Joykrishna Sarkar, Sharon Bruce, T. Kue Young

**Affiliations:** School of Public Health, University of Saskatchewan; University of Manitoba, Winnipeg, Canada; University of Manitoba, Winnipeg, Canada; University of Toronto, Toronto, Canada

## Abstract

**Introduction:**

We investigated ethnic and geographic variations in major chronic diseases and risk factors in northern Canada, an area that is undergoing rapid changes in its social, cultural, and physical environments.

**Methods:**

Self-report data were obtained from the population-based Canadian Community Health Survey in 2000-2001 and 2005-2006 for Aboriginal and non-Aboriginal respondents from the 3 regions of northern Canada: Yukon, Northwest Territories, and Nunavut. Crude prevalence estimates, adjusted odds ratios (AORs), and confidence intervals were calculated for multiple chronic diseases and risk factors.

**Results:**

The percentage of Aboriginal respondents who reported having any chronic health condition increased between the 2 cycles of data collection, but did not change for non-Aboriginal respondents. AORs for heart disease, arthritis, and asthma varied by ethnicity or region. AORs for overweight, obesity, daily smoking, regular and binge drinking, and infrequent physical/leisure activity were also substantially different for Aboriginal and non-Aboriginal respondents or among respondents from the 3 northern regions.

**Conclusion:**

The changing profile of health in northern Canada suggests a need for action on health policy about the delivery of community-based primary prevention interventions and further research about the determinants of health and health care use.

## Introduction

People in circumpolar regions of Canada are experiencing rapid changes in their social, cultural, and physical environments. Scientists are increasingly concerned about the effects of these changes on population health, particularly the health of Aboriginal populations, which includes First Nations, Inuit, and Métis groups. The increasing prevalence of "diseases of modernization," such as diabetes and high blood pressure, is already being observed (1). In northern Canada, more than half of the population is composed of Aboriginal people, primarily First Nations and Inuit, unlike most other circumpolar regions where Aboriginal people are a minority (2).

Although northern Canada is sparsely populated and communities are geographically disparate, this region has experienced rapid growth in some areas in recent years. The total population recently exceeded 100,000 (3); the largest increase occurred in the Northwest Territories (NWT) and in Nunavut. The Canadian north enjoys a higher per capita gross domestic product (GDP) than Canada as a whole, and the GDP of the NWT is about 2.5 times that of Canada's GDP, primarily attributable to increased activity associated with the extraction of natural resources (4). However, this wealth is not evenly distributed; socioeconomic indicators show that Aboriginal people in the region are disadvantaged (2).

Across Canada, Aboriginal populations experience substantial variability in living conditions and lifestyles. Some illness and death patterns at the population level are drawing increased attention. For example, the prevalence of some chronic diseases, including diabetes, heart disease, and high blood pressure, is higher among the First Nations population than among the rest of the Canadian population; the prevalence of some risk factors, such as smoking and infrequent physical activity, is also substantially higher among the First Nations population than among the rest of the Canadian population (5). Although the prevalence of many chronic diseases is lower among the Inuit than among the rest of the Canadian population, the prevalence of smoking is higher and obesity is also increasing (2). However, not enough research has been conducted that explores the variation of these chronic diseases and their risk factors within the populations of northern Canada.

## Methods

### Study design and data source

We conducted a cross-sectional analysis for 2 time periods by using data from the population-based Canadian Community Health Survey (CCHS) (http://www.statcan.gc.ca/concepts/hs-es/index-eng.htm). CCHS is a national health survey conducted by Statistics Canada to provide regular and timely information about health status, determinants of health, and health system use for 136 health regions in Canada, including the 3 regions of northern Canada: Yukon, NWT, and Nunavut. The survey covers approximately 98% of the Canadian population aged 12 years or older; excluded are people living on Indian reserves and other government-owned land, institutional residents, and full-time members of the Canadian Forces.

The first cycle of data collection, cycle 1.1, occurred over a 14-month period from September 1, 2000, through November 2, 2001. A total of 131,535 respondents participated in the study, and the overall response rate was 84.7%. Since the first cycle, data collection has occurred at regular 2-year intervals (22). The most recent available data are from cycle 3.1, which were collected from January 4, 2005, through January 8, 2006. The number of respondents was 132,947, and the overall response rate was 78.9% (22). In northern Canada, the response rate for cycle 3.1 was 81.6% in Yukon, 81.7% in NWT, and 87.7% in Nunavut (24).

All respondents to cycle 1.1 or cycle 3.1 who self-reported Yukon, NWT, or Nunavat as their region of residence were included in the study. We focused on the adult population, so people younger than aged 20 years were excluded.

The University of Manitoba Health Research Ethics Board approved this research. We obtained permission to access the data from Statistics Canada and conducted all analyses within the secure environment of the Statistics Canada Research Data Centre located at the University of Manitoba.

### Study measures

We selected the outcome measures for investigation from the following CCHS modules: chronic conditions, height and weight, smoking, alcohol use, and physical activity. Module content and question wording changed over time; therefore, we carefully selected questions common to both survey cycles.

In the chronic disease module, respondents were asked if they had ever been diagnosed with each of several chronic health conditions by a health professional. In the first cycle, only physical health conditions were included. In the second cycle, both physical and mental health conditions were investigated. Dichotomous response variables (ie, presence/absence) that were common to both survey cycles were defined for the following conditions: asthma, arthritis/rheumatism, bowel disorders, cancer, diabetes, emphysema/chronic obstructive pulmonary disease (COPD), heart disease, high blood pressure, and stroke. In addition, a dichotomous variable developed by CCHS methodologists to indicate the presence of any chronic condition was included. The following conditions were used to define this indicator of health status: food allergies, other allergies, asthma, fibromyalgia, arthritis/rheumatism, high blood pressure, back problems, migraine headaches, chronic bronchitis, emphysema, COPD, diabetes, epilepsy, heart disease, cancer, stomach or intestinal ulcers, urinary incontinence, bowel disorders, cataracts, glaucoma, thyroid condition, chronic fatigue syndrome, multiple chemical sensitivities, and any other long-term condition diagnosed by a health professional.

Body mass index (BMI) was calculated from weight and height data. Two categories were defined according to Canadian guidelines (6): 1) overweight, BMI between 25.0 and 29.9; and 2) obese, BMI of 30.0 or more.

In the smoking module, respondents were asked whether they smoked daily, occasionally, or not at all. Using these results, we formed a single dichotomous variable of daily smoking (yes/no).

We used questions about the frequency of alcohol consumption to define the following mutually exclusive categories of drinkers: regular, occasional, former, and never. Regular drinkers were respondents who had in the past 12 months, consumed alcoholic beverages at least once a month. Occasional drinkers were defined as respondents who consumed alcohol less than once a month in the past 12 months. Former drinkers were respondents who had not consumed alcoholic beverages in the last 12 months, but who reported having ever consumed alcohol. The data were used to form a single dichotomous variable defined as regular drinker (yes/no). To define binge drinking, respondents were asked about the quantity of alcohol consumed, specifically, whether they had ever consumed 5 or more drinks on a single occasion (7).

We calculated the average monthly frequency of all physical activities lasting 15 minutes or more by using the responses about physical activities lasting 15 minutes or more for the 3-month period before the date of the interview. Respondents were classified as participating in regular physical activity if the average monthly frequency of physical activity was 12 occasions or more (ie, 3 or more times per week), occasional physical activity if the average monthly frequency was between 4 and 11 occasions, and infrequent physical activity if the average monthly frequency was fewer than 4 occasions. These response categories were grouped to form a dichotomous variable with the categories of infrequent and regular/occasional. Levels of leisure physical activity were derived from each respondent's total daily energy expenditure during leisure time activities (8). Respondents were initially categorized as active (≥3.0 kcal/kg/day), moderate (1.5-2.99 kcal/kg/day), or inactive (0-1.49 kcal/kg/day); the former 2 categories were combined. Leisure physical activities included individual pursuits such as walking, running, swimming, fishing, and gardening and also team sports such as ice hockey, basketball, volleyball, and soccer.

Aboriginal and non-Aboriginal respondents were distinguished by a question about cultural or racial background. In the CCHS, the term Aboriginal encompasses North American Indians, Métis, and Inuit/Eskimo groups.

### Statistical analyses

Data from cycles 1.1 and 3.1 were analyzed separately. Survey weights were used in all analyses; these weights ensure that the final estimates are representative of the total population within each of the 3 regions of northern Canada.

The percentage of respondents reporting each of the chronic diseases and risk factors was calculated, along with 95% confidence intervals. Weighted multiple logistic regression was used to test for an association between each chronic disease or risk factor and ethnicity and region. Each model included the covariates of age group (20-44 y, 45-54 y, 55-64 y, 65-74 y, and ≥75 y) and sex. The reference category for ethnicity was non-Aboriginal, and for region, it was NWT. The latter was chosen because geographically this region is between the other 2 northern regions, and its population contains approximately the same proportion of Aboriginal and non-Aboriginal residents.

We used a bootstrap method to calculate 95% confidence intervals (CIs) for the estimated adjusted odds ratios (AORs) and *P* values (9-11). The bootstrap method randomly samples with replacement from the original set of observations to obtain a sampling distribution for a population parameter (eg, AOR). SAS software, version 9.1 (SAS Institute, Inc, Cary, North Carolina), was used to conduct all analyses. We used a SAS macro that was developed by methodologists at Statistics Canada to calculate the bootstrap 95% CIs; these were based on 500 samples, as recommended by the macro developers.

## Results

There were 2,074 respondents from northern Canada in cycle 1.1 and 2,166 respondents in cycle 3.1. Approximately one-quarter (26.7%) of respondents in cycle 1.1 were from Nunavut, one-third (33.6%) were from Yukon, and the remainder were from NWT (39.8%) (Table 1). The geographic distribution of respondents was similar in cycle 3.1. Approximately 40% of respondents in each cycle were of Aboriginal ethnicity, although ethnic composition varied across the 3 regions.

In the first cycle, the percentage of Aboriginal respondents who reported having any chronic health condition was lower compared with the percentage of non-Aboriginal respondents (Table 2). However, there was a large increase between the 2 cycles for Aboriginal respondents (8.8 percentage point difference), but not for non-Aboriginal respondents (0.6 percentage point difference). This increase was observed for both younger (20-54 years) and older (≥55 years) age groups (Figure). In the first cycle, Yukon respondents reported the highest crude prevalence of any chronic condition, and prevalence estimates were similar for NWT and Nunavut respondents. Prevalence estimates for any chronic condition were similar for all regions between cycles 1.1 and 3.1, with the exception of NWT, where an increase was observed.

**Figure 1:**
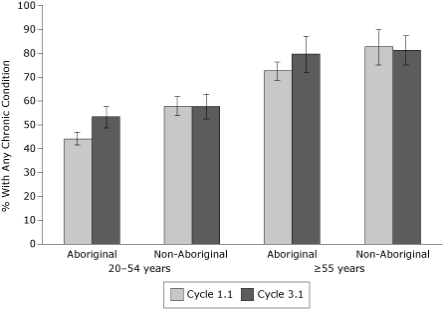
Percentages of any chronic condition by ethnicity and age, Canadian Community Health Survey, cycle 1.1 (2000-2001) and cycle 3.1 (2005-2006) (error bar = 95% confidence interval).

**Table:** 

**Cycle 1.1**		
**Ethnicity/Age Group**	**Percentage**	**95% Confidence Interval**
**Aboriginal**		
20-54 years	44	42-47
≥55 years	73	69-76
**Non-Aboriginal**		
20-54 years	58	54-62
≥55 years	83	75-90
**Cycle 3.1**		
**Aboriginal**		
20-54 years	53	49-58
≥55 years	80	72-87
**Non-Aboriginal**		
20-54 years	58	53-63
≥55 years	81	75-87

The prevalence of all of the investigated chronic diseases was similar in cycles 1.1 and 3.1. Arthritis was the most common chronic condition reported; it was reported by 13% of northern Canada respondents, followed by high blood pressure (approximately 10%) and asthma (approximately 8%) ( Table 2). Prevalence estimates were slightly higher for respondents from Yukon than for respondents from the other 2 regions. Data for some diseases and risk factors were not included in our analyses because of low frequencies. Specifically, data for bowel disorders, cancer, emphysema/COPD, and stroke were not reported.

Approximately one-quarter of Aboriginal and non-Aboriginal respondents were classified as overweight, and approximately 20% were classified as obese in Cycle 1.1 (Table 2). Between the 2 cycles of data collection, there was an increase (5.3 percentage point difference) in overweight for non-Aboriginal respondents, but not for Aboriginal respondents. There was an increase in obesity for Aboriginal (5.2 percentage point difference) and non-Aboriginal (2.6 percentage point difference) respondents. Approximately one-third of NWT respondents were classified as overweight in cycle 1.1; prevalence was lower in the other 2 regions.

More than one-half of Aboriginal respondents were identified as daily smokers in both cycle 1.1 (52.5%) and cycle 3.1 (50.2%), compared with less than one-third (29.9%) of non-Aboriginal respondents in the first cycle and less than one-quarter (23.5%) of the latter group in the second cycle. Daily smoking rates were highest in Nunavut (50.2%) and lowest in Yukon (27.8%) in cycle 3.1. Regular drinking and binge drinking were more common among non-Aboriginal and Yukon respondents. Higher prevalence of infrequent physical activity and inactivity during leisure time was observed among non-Aboriginal and Nunavut respondents (Table 2).

The adjusted odds of having any chronic health condition were significantly lower and the odds of heart disease were significantly higher for Aboriginal respondents in cycle 1.1, but the AORs for these 2 variables were not significant (any chronic condition, *P* = .61; heart disease, *P* = .78) in cycle 3.1 (Table 3). Among Aboriginal respondents, in both survey cycles, odds of daily smoking and infrequent physical activity were higher than for non-Aboriginal respondents, but odds of regular drinking and binge drinking were lower. Moreover, the ORs for daily smoking and infrequent physical activity were larger in cycle 3.1 than in cycle 1.1.

By region, the odds of having any chronic condition were highest for Yukon respondents in cycle 1.1 (Table 3). The odds of arthritis were lowest for Nunavut respondents in cycle 1.1. The odds of asthma were lower for Nunavut respondents in both cycles. There were no regional differences for other chronic diseases.

For Yukon respondents, although there were a number of significant differences in the AORs for risk factors, those for obesity and inactivity during leisure time (lower odds) were observed in both cycles (Table 3). For Nunavut respondents, the odds of daily smoking were higher than for respondents from the other regions in both cycles, and the odds of regular drinking were significantly lower in cycle 1.1.

## Discussion

These population-based health survey data suggest that chronic diseases and risk factors are not uniformly distributed across ethnic groups and geographic regions of northern Canada and that some negative shifts have occurred in their distribution in a relatively short period of time.

Some of the findings of this study are consistent with previous research comparing Aboriginal and non-Aboriginal populations in southern Canada or in other countries, but others are not. For example, smoking rates are reported elsewhere to be higher among Aboriginals compared with non-Aboriginals (8,12). A previous study that compared chronic disease and risk factors between northern and southern Canadian populations found that the Aboriginals had a higher prevalence of overweight and obesity, daily smoking, and physical inactivity than the non-Aboriginals (13). However, we found that these risk factors among northern Canadian populations varied substantially. In addition, with respect to overweight, recent studies suggest that Canadian Aboriginals have substantially higher rates of overweight than non-Aboriginals with self-reported white ethnicity (14,15), which contradicts our findings. The difference may arise, in part, because the non-Aboriginal group in this study was not limited to whites.

Overweight, obesity, smoking, binge drinking, and inactivity threaten the long-term health of northern Canadian populations. Excess weight and smoking increase the risk of cardiovascular disease (16), smoking is associated with an increase in certain forms of cancer, and excess weight can also contribute to the development of chronic musculoskeletal conditions.

A recent study, which reviewed the literature on self-reported cancer screening behaviors and race, found greater bias in self-reported behaviors for racial and ethnic minority groups, including North American Indians; it suggested that a variety of factors, including motivation, cognition (ie, health literacy and knowledge of screening techniques), and the health care environment, may contribute to bias in estimates from self-reported data (17). Chronic disease prevalence estimates may not agree with estimates obtained from other population-based sources. For example, 1 study found that agreement between survey and administrative data was highest for diabetes and high blood pressure, but much lower for arthritis and heart disease (18).

There are several limitations to our study. We relied on self-reported data, which may have underestimated the prevalence of overweight, obesity, smoking, and binge drinking and overestimated the prevalence of physical activity (19). Health care services and screening programs for Aboriginal people in northern Canada may be less accessible than for non-Aboriginal people; this may result in biased self-reporting about chronic diseases because the survey questions in this study focused on conditions diagnosed by a health professional (8). Measures of overweight and obesity developed in European populations may not be appropriate for Aboriginal populations (20), especially the Inuit, whose ancestors came from Asia (21); in general, people of Asian descent have more risk factors and higher body fat than people of European descent with the same BMI. Leisure time physical activity does not include activities performed at work (eg, work-related labor activity), at school (eg, physical education class), or at home (eg, household chores). The measure of any chronic condition used in this study provides an overall indicator of health status but obscures changes in individual conditions; an increase in this indicator does not necessarily represent an increase in the prevalence of all chronic conditions. The CCHS covers 90% of the Yukon population and 97% of the NWT population, but only 71% of the Nunavut population (22) (data were only collected in the 10 largest communities in Nunavut). Thus, the results for the latter region may not be as representative of the entire population as results for the other 2 regions. Finally, we used cross-sectional data to estimate change in chronic diseases and risk factors instead of using repeated measurements on the same cohort of respondents to study the progression of health.

Although these survey results can provide snapshots of health status and its determinants, further analyses and other population-based data sources are needed to obtain a more complete picture of chronic disease in northern Canada, its association with health care use, and the effect of community-based interventions on changes in health status. Not enough information exists about different Aboriginal groups, particularly Inuit and First Nations groups, who do not have identical disease burden or risk factor distribution. Data from multiple cycles of the CCHS may be combined to achieve sufficient sample size to enable comparisons of First Nations and Inuit groups within northern Canada. Administrative data, including hospital and physician records, are 1 source of routinely collected data that can be used to further study ethnic and regional variations in chronic disease and their association with health care use (18). The Aboriginal Peoples Survey, which was initiated by the federal government in 2001, addresses gaps in other sources, including the lack of data about some determinants of Aboriginal health (eg, housing, employment, residential mobility). More health policies are needed that are culturally sensitive and that focus on the delivery of preventive interventions (23).

The findings of our study, which suggest an overall increasing prevalence of chronic disease in Aboriginal populations and negative shifts in the distribution of risk factors, underscore the importance of community-based primary preventive interventions at the community and primary care level in northern communities.

## Figures and Tables

**Table 1:** Characteristics of Canadian Community Health Survey Respondents, Cycle 1.1 (2000-2001) and Cycle 3.1 (2005-2006)

Cycle/Characteristics	Yukon Respondents No. (%)	NWT Respondents No. (%)	Nunavut Respondents No. (%)	Total Respondents No. (%)
**Cycle 1.1**				
**Total**	696 (100)	825 (100)	553 (100)	2,074 (100)
**Ethnicity^a^**				
Aboriginal	120 (17.2)	382 (46.3)	364 (65.8)	866 (41.8)
Non-Aboriginal	560 (80.5)	429 (52.0)	185 (33.5)	1,174 (56.6)
**Sex**				
Male	324 (46.6)	410 (49.7)	295 (53.4)	1,029 (49.6)
Female	372 (53.5)	415 (50.3)	258 (46.7)	1,045 (50.4)
**Age, y**				
20-34	191 (27.4)	299 (36.2)	274 (49.6)	764 (36.8)
35-44	194 (27.9)	237 (28.7)	138 (25.0)	569 (27.4)
45-54	167 (24.0)	166 (20.1)	68 (12.3)	401 (19.3)
≥55	144 (20.7)	123 (14.9)	73 (13.2)	340 (16.4)
**Cycle 3.1**				
**Total**	731 (100)	830 (100)	605 (100)	2,166 (100)
**Ethnicity^a^**				
Aboriginal	132 (18.1)	305 (36.7)	373 (61.7)	810 (37.4)
Non-Aboriginal	590 (80.7)	522 (62.9)	228 (37.7)	1,340 (61.9)
**Sex**				
Male	338 (46.2)	406 (48.9)	328 (54.2)	1,072 (49.5)
Female	393 (53.8)	424 (51.1)	277 (45.8)	1,094 (50.5)
**Age, y**				
20-34	176 (24.1)	314 (37.8)	281 (46.5)	771 (35.6)
35-44	167 (22.9)	196 (23.6)	155 (25.6)	518 (23.9)
45-54	190 (26.0)	152 (18.3)	92 (15.2)	434 (20.0)
≥55	198 (27.1)	168 (20.2)	77 (12.7)	443 (20.5)

Abbreviation: NWT, Northwest Territories.

a Percentages may not total 100 because of missing data.

**Table 2:** Crude Percentages (95% CIs) for Major Chronic Diseases and Risk Factors by Ethnicity and Region, Canadian Community Health Survey, Cycle 1.1 (2000-2001) and Cycle 3.1 (2005-2006)

Ethnicity or Region/Risk Factor or Chronic Condition of Respondents^a^	Cycle 1.1 RespondentsCrude % (95% CI)	Cycle 3.1 RespondentsCrude % (95% CI)
**Aboriginal**		
Any chronic condition	48.5 (46.0-51.1)	57.3 (53.2-61.3)
Arthritis	11.6 (10.0-13.2)	11.5 (8.8-14.1)
Asthma	6.1 (4.9-7.4)	6.3 (4.0-8.5)
Diabetes	3.2 (2.1-4.3)	3.8 (2.2-5.4)
Heart disease	5.1 (3.9-6.2)	2.7 (1.3-4.1)
High blood pressure	9.4 (7.8-11.1)	10.9 (8.3-13.5)
Overweight	28.6 (26.1-31.0)	26.2 (22.1-30.2)
Obese	20.2 (18.1-22.4)	25.4 (20.5-30.2)
Daily smoker	52.5 (50.0-55.0)	50.2 (45.7-54.8)
Regular drinker	44.4 (42.5-46.3)	51.6 (46.5-56.7)
Binge drinker	17.7 (16.4-19.0)	22.9 (19.7-26.1)
Infrequent physical activity	28.3 (26.5-30.1)	29.2 (24.6- 33.8)
Inactive leisure time	52.7 (50.5-55.0)	58.0(53.0-63.1)
**Non-Aboriginal**		
Any chronic condition	61.6 (58.1-65.1)	62.2 (57.8-66.5)
Arthritis	13.7 (12.1-15.4)	14.9 (12.5-17.3)
Asthma	8.5 (6.2-10.7)	8.6 (6.6-10.5)
Diabetes	3.1 (2.3-3.9)	3.8 (2.5-5.0)
Heart disease	3.3 (2.1-4.4)	2.7 (1.9-3.6)
High blood pressure	9.4 (7.6-11.2)	11.3 (9.3-13.2)
Overweight	28.7 (26.0-31.5)	34.0 (30.9-37.1)
Obese	18.5 (15.9-21.0)	21.1 (18.3-23.9)
Daily smoker	29.9 (26.5-33.3)	23.5 (20.1-26.9)
Regular drinker	65.3 (62.0-68.5)	66.8 (63.0-70.5)
Binge drinker	36.1 (33.0-39.2)	38.5 (34.8-42.2)
Infrequent physical activity	19.2 (16.5-22.0)	18.5 (15.9-21.1)
Inactive leisure time	42.6 (39.2-45.9)	45.8 (41.5-50.0)
**Yukon**		
Any chronic condition	65.3 (60.6-70.1)	65.4 (61.5-69.4)
Arthritis	17.2 (14.4-20.0)	16.0 (13.1-18.9)
Asthma	8.9 (6.4-11.4)	8.4 (6.1-10.7)
Diabetes	3.8 (2.3-5.2)	4.7 (3.1-6.4)
Heart disease	4.6 (2.7-6.5)	3.1 (1.8-4.4)
High blood pressure	10.0 (7.9-12.2)	12.9 (10.5-15.3)
Overweight	26.1 (22.6-29.5)	29.9 (25.5-34.2)
Obese	15.2 (11.5-18.8)	18.4 (14.5-22.2)
Daily smoker	28.2 (23.7-32.7)	27.8 (22.9-32.7)
Regular drinker	65.4 (60.2-70.6)	65.6 (60.2-71.1)
Binge drinker	36.4 (31.8-41.0)	36.9 (32.2-41.6)
Infrequent physical activity	14.4 (11.1-17.7)	19.5 (16.0-23.1)
Inactive leisure time	35.6 (31.3-40.0)	44.2 (39.5-49.0)
**Nunavut**		
Any chronic condition	48.8 (45.2-52.5)	50.0 (43.3-56.7)
Arthritis	7.5 (5.6-9.3)	8.3 (4.5-12.1)
Asthma	4.4 (3.7-5.0)	3.5 (1.1-5.8)
Diabetes	2.0 (1.3-2.6)	0.8 (0.0-2.0)
Heart disease	3.6 (1.9-5.3)	1.5 (0.2-2.8)
High blood pressure	7.7 (6.3-9.2)	8.6 (6.5-10.8)
Overweight	28.6 (25.7-31.5)	29.8 (25.2-34.5)
Obese	21.4 (18.3-24.4)	26.1 (22.8-29.4)
Daily smoker	54.0 (49.7-58.3)	50.2 (44.2-56.2)
Regular drinker	40.0 (36.8-43.1)	59.4 (54.5-64.3)
Binge drinker	24.3 (21.7-27.0)	28.6 (23.0-34.1)
Infrequent physical activity	29.0 (25.2-32.7)	29.5 (22.8-36.1)
Inactive leisure time	29.0 (48.7-55.9)	57.0 (50.7-63.2)
**NWT**		
Any chronic condition	52.6 (48.6-56.5)	60.4 (54.4-66.3)
Arthritis	12.8 (11.5-14.0)	14.0 (11.2-16.8)
Asthma	8.1 (5.0-11.3)	8.9 (6.5-11.2)
Diabetes	3.2 (2.2-4.3)	4.1 (2.4-5.8)
Heart disease	3.8 (2.7-4.8)	2.8 (1.7-3.9)
High blood pressure	9.6 (7.3-11.9)	10.6 (8.1-13.1)
Overweight	31.0 (28.0-34.0)	33.0 (29.5-36.5)
Obese	20.8 (18.4-23.2)	24.6 (20.5-28.7)
Daily smoker	39.1 (36.6-41.6)	30.5 (26.3-34.6)
Regular drinker	58.5 (55.6-61.4)	58.9 (54.2-63.6)
Binge drinker	23.9 (21.1-26.8)	31.5 (27.4-35.6)
Infrequent physical activity	27.2 (24.3-30.0)	22.3 (18.7-25.9)
Inactive leisure time	52.8 (50.5-55.1)	52.5 (47.9-57.2)

Abbreviations: CI, confidence interval; NWT, Northwest Territories.

a See "Methods" section for a description of how risk factors were defined.

**Table 3:** Adjusted Odds Ratios (AORs) for Major Chronic Diseases and Risk Factors by Ethnicity and Region, Canadian Community Health Survey, Cycle 1.1 (2000-2001) and Cycle 3.1 (2005-2006)

Ethnicity or Region/Risk Factor or Chronic Disease of Respondents^a^	Cycle 1.1		Cycle 3.1	

	AOR (95% CI)^b^,^c^	*P* Value	AOR (95% CI)^b^,^c^	*P* Value
**Aboriginal**				
Any chronic condition	0.70 (0.58-0.84)	<.001	0.93 (0.71-1.22)	.61
Arthritis	1.07 (0.84-1.37)	.60	0.80 (0.55-1.16)	.24
Asthma	0.75 (0.54-1.04)	.09	0.73 (0.45-1.17)	.19
Diabetes	1.52 (0.85-2.70)	.16	1.38 (0.70-2.73)	.35
Heart disease	2.15 (1.19-3.91)	.01	1.10 (0.55-2.23)	.78
High blood pressure	1.25 (0.85-1.85)	.26	1.18 (0.78-1.78)	.42
Overweight	1.02 (0.81-1.28)	.86	0.67 (0.51-0.90)	.01
Obese	1.07 (0.86-1.35)	.53	1.18 (0.84-1.67)	.34
Daily smoker	2.19 (1.73-2.78)	<.001	2.99 (2.29-3.91)	<.001
Regular drinker	0.51 (0.43- 0.60)	<.001	0.53 (0.38-0.74)	<.001
Binge drinker	0.43 (0.35-0.52)	<.001	0.45 (0.34-0.61)	<.001
Infrequent physical activity	1.28 (1.01-1.63)	.05	1.62 (1.22-2.14)	<.001
Inactive leisure time	1.21 (0.98-1.49)	.08	1.44 (1.06-1.96)	.02
**Yukon**				
Any chronic condition	1.46 (1.07-1.98)	.02	1.10 (0.78-1.54)	.59
Arthritis	1.32 (0.98-1.77)	.07	0.87 (0.58-1.31)	.51
Asthma	1.02 (0.56-1.83)	.96	0.88 (0.56-1.37)	.57
Diabetes	1.14 (0.60-2.16)	.68	1.00 (0.51-1.93)	.99
Heart disease	1.43 (0.75-2.73)	.28	0.85 (0.43-1.65)	.62
High blood pressure	0.93 (0.59-1.46)	.76	1.04 (0.71-1.54)	.83
Overweight	0.76 (0.58-0.99)	.04	0.78 (0.58-1.06)	.11
Obese	0.66 (0.47-0.93)	.02	0.68 (0.48-0.96)	.03
Daily smoker	0.81 (0.62-1.06)	.13	1.14 (0.84-1.56)	.40
Regular drinker	1.15 (0.86-1.55)	.35	1.32 (0.96-1.82)	.09
Binge drinker	1.38 (1.06-1.80)	.02	1.04 (0.78-1.38)	.80
Infrequent physical activity	0.46 (0.33-0.64)	<.001	0.89 (0.65-1.22)	.48
Inactive leisure time	0.50 (0.40-0.63)	<.001	0.74 (0.55-0.99)	.04
**Nunavut**				
Any chronic condition	0.94 (0.75-1.19)	.61	0.68 (0.46-1.01)	.06
Arthritis	0.56 (0.41-0.77)	<.001	0.63 (0.33-1.20)	.16
Asthma	0.53 (0.33-0.85)	.01	0.38 (0.15-0.99)	.05
Diabetes	0.61 (0.36-1.03)	.07	0.20 (0.10-6.57)	.36
Heart disease	0.91 (0.49-1.69)	.76	0.58 (0.18-1.81)	.34
High blood pressure	0.80 (0.55-1.18)	.26	0.86 (0.55-1.35)	.52
Overweight	0.88 (0.71-1.09)	.25	0.99 (0.74-1.32)	.93
Obese	1.05 (0.82-1.35)	.69	1.07 (0.77-1.47)	.69
Daily smoker	1.66 (1.35-2.03)	<.001	1.74 (1.29-2.34)	<.001
Regular drinker	0.48 (0.40-0.58)	<.001	1.18 (0.87-1.62)	.29
Binge drinker	1.17 (0.94-1.46)	.15	1.10 (0.74-1.63)	.64
Infrequent physical activity	1.08 (0.85-1.37)	.52	1.32 (0.88-1.96)	.18
Inactive leisure time	0.97 (0.81-1.16)	.73	1.09 (0.80-1.49)	.58

a See "Methods" section for a description of how risk factors were defined.

b AORs are calculated for the population in all 3 territories and are adjusted for age, sex, and region; the non-Aboriginal population is the reference group.

c AORs are calculated for the population in all 3 territories and are adjusted for age, sex, and ethnicity; the Northwest Territories (NWT) region is the reference group.
